# Prevalence and Factors Associated with Back Pain among Patients Undergoing Spinal Anesthesia at the University of Gondar Comprehensive and Specialized Hospital, North West Ethiopia: An Institutional Based Cross-Sectional Study

**DOI:** 10.1155/2021/6654321

**Published:** 2021-01-25

**Authors:** Tadael Gudayu Zeleke, Abraham Tarekegn Mersha, Nigussie Simeneh Endalew, Yonas Admasu Ferede

**Affiliations:** Department of Anesthesiology and Critical Care, School of Medicine, College of Medicine and Health Science, University of Gondar, Gondar, Ethiopia

## Abstract

**Background:**

Back pain is often reported as a common complaint after surgery. Many studies showed that the prevalence of back pain after spinal anesthesia is high and its magnitude is considerable in developing countries. It is highly related to reduced quality of life, loss of work productivity, burden of health care costs, and satisfaction regarding health care service; therefore, measures should be taken to reduce or prevented postspinal back. The aim of this cross-sectional study was to assess the prevalence and factors associated with back pain among patients undergoing spinal anesthesia at the University of Gondar Comprehensive and Specialized Hospital, Northwest Ethiopia. *Methodology*. An institutional based cross-sectional study was conducted from March to May 2020. A total of 215 participants were enrolled in this study. A convenience sampling technique was used to get the study participants. Both univariable and multivariable logistic regression were used to identify factors associated with postspinal back pain. Variables with a *p* value less than <0.2 in the bivariable analysis were fitted into the multivariable analysis. In the multivariable analysis, a variable with a *p* value of <0.05 was considered statistically significant.

**Results:**

The overall prevalence of postspinal back pain was 40.5% (95% CI: 34.0, 47.4). Being overweight (AOR = 3.8; 95% CI: 1.47, 9.96) and obese (AOR = 4.9; 95% CI: 1.19, 20.4), using big spinal needles (AOR = 5.9; 95% CI: 1.04, 33.4), two attempts of lumbar puncture (AOR = 5.5; 95% CI: 1.74, 17.59), more than three attempts of lumbar puncture (AOR = 4.9; 95% CI: 1.63, 15.2), and the number of bone contacts during spinal anesthesia procedure (AOR = 3.1; 95% CI: 1.14, 8.45) were positively associated with postspinal back pain. *Conclusion and Recommendation*. The overall incidence of back pain is high. Body mass index, size of spinal needle, number of attempts, and number of bone contacts are significantly associated with the incidence of back pain following spinal anesthesia. Thus, it is better to minimize the number of lumbar puncture attempts and bone contacts during spinal anesthesia to reduce postspinal back pain. In addition, using smaller size spinal needle is a good choice.

## 1. Introduction

Spinal anesthesia (SA) is the most commonly preferred and widely used anesthesia technique in surgeries like lower extremity surgery, anorectal, urologic, obstetric, and gynecologic interventions and lower abdominal procedures. [1, 2] Even though spinal anesthesia is the preferred technique, it has a lot of complications like postspinal back pain (PSBP) compared to general anesthesia (GA). [2–6].

Back pain is a worldwide health problem affecting 50% and 80% of people at some time in their lives and it is a major physical and economical burden for the individual and the society [7–10]. The lifetime incidence of nonspecific low back pain is more than 84%, the incidence of chronic low back pain is about 23%, and 11-12% of the population suffered disability with this pain [11].

Regardless of the anesthetic technique, postoperative low back pain is often reported as a common complaint after SA but the association between anesthetic technique and back pain is still unclear [12]. Postspinal back pain (PSBP) is usually a mild type of pain and it manifests during the first two to six hours (hrs) after the surgical procedure, when the local anesthetics wear off in most people, and lasts only for a few days [5]. Rarely, the pain may persist for some weeks and becomes permanent because of nerve injury during spinal needle insertion [13, 14].

Studies showed the incidence of back pain ranges 10.7–12.3% after spinal anesthesia [5, 15, 16]. A study conducted in Ethiopia reported that 38.0%, 29.9%, and 16.0% of patients suffered PSBP in the 1^st^, 2^nd^, and 3^rd^ postoperative days after SA, respectively [4]. Another study done in Asella, Ethiopia, showed that patients suffered backache in the postoperative 1^st^, 2^nd^, and 3^rd^ days and 4^th^ week after spinal anesthesia in surgical procedures with 38.0, 29.9, 16.0, and 31.6% respectively [17]. Fear of back pain after SA is one reason for patient refusal of this type of anesthesia and it accounts for a refusal rate of 13.4% [16, 18].

In a study done in Chicago, USA, 9-10% of the study participants had postoperative back pain after SA [19]. On the other hand, in a multicenter prospective study in Europe, back pain was the leading complaint among other postlumbar puncture complaints with an incidence of 17% [20]. A higher prevalence rate, 40%, of back pain after spinal anesthesia was found in a prospective observational study in Germany, on 112 patients [21]. In addition, studies done in Turkey and China showed that PSBA occurred in 29.3% of patients [1] and 39% at the first, 37% at the second, and 31% at the third postoperative days [22] after SA, respectively.

Back pain remains the determinant factor for patient satisfaction after spinal anesthesia [23]. Apart from uncomfortable experience, untreated back pain cause side effects like aggravating wound pain by stretching incisions, increasing intracranial and intraocular pressure, increasing expenditure of cardiac and systemic energy, and increasing tissue oxygen demand and delayed discharge from postanesthetic care and also from hospitals [16]. This may be very deleterious in patients particularly those with impaired cardiovascular reserve or a limited respiratory capacity [4]. Overall, in long-term effect, postspinal back pain may be related to reduced quality of life, sickness, absence, loss of work productivity, and high health care costs [24].

Giving concise and detailed information regarding postoperative back pain after spinal anesthesia during the informed consent might improve satisfaction with the anesthetic procedure. [22] Therefore, the aim of this study is to assess the prevalence and factors associated with back pain among patients undergoing spinal anesthesia at the University of Gondar Comprehensive Specialized Hospital.

## 2. Materials and Methods

### 2.1. Study Setting and Population

An institutional based cross-sectional study was conducted at the University of Gondar Comprehensive Specialized Hospital from March to May 2020. The University of Gondar Comprehensive and Specialized Hospital is one of the biggest governmental teaching hospitals which is located in Amhara region, Central Gondar Zone, about 738 km away from the capital city, Addis Ababa, and 230 km from Ethiopia-Sudan border in the North west of Ethiopia. It has been giving services for millions of patients in the region.

All patients scheduled for elective or emergency surgery under spinal anesthesia during data collection period were enrolled in this study whereas patients with preexisting back pain, patients <16 years, traumatic deformity of the spine or congenital abnormalities of the lumbar spine, impaired cognitive ability, and patients undergoing combined spinal and epidural anesthesia were excluded from the study.

### 2.2. Sample Size and Sampling Procedure

Single population proportion formula was used to determine the sample size. It was calculated by considering a 95% confidence interval, a 5% margin of error, and 38.0% as a proportion of incidence of postspinal back pain in the first day based on a study conducted in Asella, Ethiopia. [17] Then, a sample size of 362 patients was obtained. However, we used a correction formula since the study population is less than 10,000. Finally, a size of 215 was used considering a 10% nonresponse rate.

The convenience sampling technique was used in all patients undergoing surgical operation under spinal anesthesia at the University of Gondar Comprehensive Specialized Hospital until the required sample size was reached.

### 2.3. Operational Definition

#### 2.3.1. Postspinal Back Pain

The symptom of pricking sensation or local tenderness at the site of needle insertion is characterized by tenderness without radiating pain to the buttock or/and to lower extremities and no neurological findings [16, 18, 22, 25–28].

#### 2.3.2. Visual Analogue Scale (VAS)

It is a method of pain assessment tool determined by the patient making a mark of their pain intensity on a line which is 100 millimeters long. It is a horizontal line with “no pain” at one end to “worst possible pain” at the other end of the line. It is a valid pain assessment tool [29].

No pain------------------worst imaginable pain.

In a line of 100 mm VAS rating, 0 to 4 mm can be considered no pain; 5 to 44 mm, mild pain; 45 to 74 mm, moderate pain; and 75 to 100 mm, severe pain [30, 31].

#### 2.3.3. Angle of Lumbar Puncture

The angle of lumbar puncture is the angle of needle bevels' with respect to spinal ligaments which is perpendicular or parallel to the fibers of supraspinous and interspinous ligaments [1].

#### 2.3.4. Number of Punctures

It is the number procedures starting from the introduction of the spinal needle and ending with the removal of the stylet with anticipation of CSF flashback or the number of inserting the spinal needle through the soft tissues ligaments to perform subarachnoid block [32–34].

#### 2.3.5. Number of Bone Contacts

It is the number of contacts of bone structures during an attempt to perform subarachnoid block [1].

#### 2.3.6. Spinal Needle Gauges

Spinal needles of size from 23 to 29 G are small needle gauges whereas spinal needle gauges of size from 18 to 22 G are labeled as big needle gauges [35, 36].

#### 2.3.7. Overweight and Obesity

They are defined as abnormal or excessive fat accumulation that presents a risk to health. A body mass index (BMI) over 25 to 29.9 is considered overweight, and 30 or above is obese [37].

### 2.4. Data Collection Procedures

Data was collected by chart review, patient interview, and through observation using a semistructured questionnaire prepared from different literature. Patients were asked whether they felt local tenderness or pain at the site of needle injection site or not. The patients who had felt pain were asked to mark on 100 mm horizontal line pain assessment tool (VAS score tool) to indicate the intensity of their back pain after the data collector gave them a detailed and adequate information. So, PSBP was assessed with VAS score tool whether they had felt pain or not in 24 hr, 48 hr, and 72 hrs postoperatively in postanesthesia care units (PACU) and wards. If the study participants felt PSBP, then they were asked to mark the level of pain and the data was considered but if they did not feel pain, the data collector observed those patients every 24 hrs until 72 hrs. The data collection procedure was continued until the estimated sample size is reached.

### 2.5. Data Quality Management

A half-day training was given to data collectors and supervisors about the data collection tool and how to get consent for the study. To ensure the quality of data, a pretest was done on 22 patients (10% of the sample size) who were not included in the main study. Then, the necessary correction was done on the questionnaire for the main study. The principal investigator and supervisor checked the collected data for completeness, accuracy, and clarity. Daily supervision and feedback were done by the principal investigator and supervisors during the entire data collection period. Finally, coding, data entry, data cleaning, and crosschecking were done before data analysis.

### 2.6. Data Processing, Analysis, and Interpretation

This study used Epi-info and SPSS version 20 for data entry and analysis. Descriptive statistical analysis such as simple frequencies, crosstab, measures of central tendency, and measures of variability was used to describe the characteristics of participants. Then, the information was presented using frequencies, summary measures, tables, and figures.

The association between dependent and independent variables were assessed by using univariable and multivariable logistic regression. Binary logistic regression was run to see the association between each independent variable and the outcome variable at a *p* value <0.2. A variable whose univariable test had a *p* value <0.2 was a candidate for multiple logistic regression along with all variables. The multivariable logistic regression model was used to determine factors associated with PSBP. Adjusted odds ratio with corresponding 95% confidence interval was computed to see the strength of association. In the multivariable analysis, a variable with a *p* value of <0.05 was considered statistically significant. Hosmer Lemeshow test was also used for checking goodness of fit.

## 3. Results

### 3.1. Sociodemographic Characteristics of the Study Participants

A total of 215 participants were involved in this study. The highest number 72 (33.5%) belongs to the age group of 26–34 years. More than two-thirds, 150 (69.8%), of the study participants were female and around one-third, 71 (33.0%), were housewives. Regarding their nutritional status, about 152 (70.7%) of participants had a BMI of 18.7–25 kg/m^2^ and 20 (9.3%) were obese (BMI > 30 kg/m^2^) (Table 1).

### 3.2. Prevalence of Postspinal Back Pain

The overall prevalence of PSBP in this study was 40.5% (95% CI: 34.0, 47.4).

**Figure 1:**
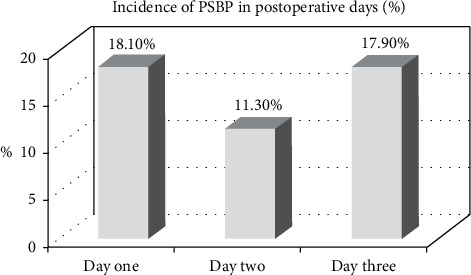
Prevalence of back pain on the three postoperative days at the University of Gondar Comprehensive and Specialized Hospital, Northwest Ethiopia, from March to May 2020 (*n* = 87).

Postoperative data showed the highest incidence found on the first day and the lowest was on the third day. On the first postoperative day, 39 (18.1%) of the study participants (*n* = 215) experienced back pain but 81.9% of them showed no postoperative backache on day one. 20 (11.3%) of the respondents (*n* = 176) experienced back pain on the second postoperative day. On the 3^rd^ postoperative day, 28 (17.9%) of the participants (*n* = 156) experienced back pain (Figure 1**).**

**Figure 2:**
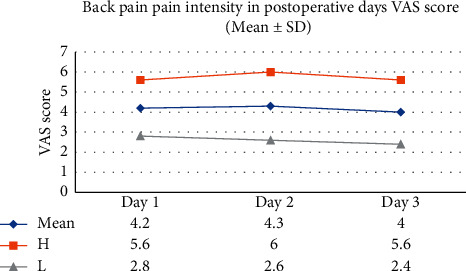
Severity of back pain on the three postoperative days at the University of Gondar Comprehensive and Specialized Hospital, North West Ethiopia, from March to May 2020 (*n* = 87).

The mean severity of back pain VAS score was 4.2 ± 1.5 SD considering the total of patients who had developed back pain (*n* = 87). The mean intensity of the pain VAS score was 4.2 ± 1.4 SD in the first postoperative day (*n* = 39), 4.3 ± 1.7 SD in the second postoperative day (*n* = 20), and 4.0 ± 1.6 SD in the third postoperative day (*n* = 28) **(**Figure 2**)**.

Among the participants, the highest percentage of PSBP (80%) happened in patients who had BMI >30 kg/m^2^ and the lowest percentage (11.8%) happened in patients who had spinal anesthesia with small size needle (Table 2). According to the experience of the anesthetists, the highest proportion (45.7%) of PSBP happened in patients who have had spinal anesthesia with anesthesia students.

PSBP was found to be more common in patients whose surgical procedure took greater than one hour (47.9%) than in patients whose surgical procedure took less than one hour (34.3%). PSBP was also more common in patients who had surgery on supine and lithotomy positions (Table 2).

Both the number of attempts of lumbar puncture and the number of bone contacts during lumbar puncture had a direct relationship with the incidence of postspinal backache. An attempt of lumbar puncture ≥3 times was found to be the highest incidence of PSBP (73.8%). In addition, those participants who had a higher number of bone contacts during spinal anesthesia had developed more back pain (Table 2).

The incidence was high in patients who had spinal anesthesia at the level of lumbar puncture interspace L_2_-L_3_ (78.9%) and back pain happened higher when the angle of the needle was perpendicular (57.8%) to the skin than was parallel (33.1%) to the skin (Table 2).

### 3.3. Factors Associated with Postspinal Back Pain

Both univariable and multivariable logistic regression analyses were done to see factors associated with PSBP. Variables like body mass index (BMI), ASA status, spinal needle size, number of attempts, number of bone contacts, angle of the needle, spinal interspace, history and numbers of previous SA exposures, and the presence of skin infiltration had a p value of <0.2 in the binary logistic regression analysis.

Finally, spinal needle size, number of attempts, and the number of bone contacts were significantly associated with PSBP in the multivariable logistic regression analysis (Table 3).

Accordingly, overweight patients were fourfold more likely to develop PSBP than patients with a BMI of <25 kg/m^2^ [AOR = 3.8 (95% CI: 1.47, 9.96)]. Similarly, obese patients were 5 times more likely to develop PSBP than patients with a BMI of <25 kg/m^2^ [AOR 4.9 (95% CI: 1.19, 20.43)]. Patients who underwent surgery with big spinal needle gauges were 6 times more likely to develop PSBP than small gauge needles [AOR = 5.9 (95% CI: 1.04, 33.46)].

The number of attempts of lumbar puncture was also significantly associated with PSBP. Patients who had 2-time attempt were 4 times more likely to develop PSBP than patients who had only a single attempt [AOR = 3.6 (95% CI: 1.29, 10.42)] and patients who had more than 3-time attempt also were 5 times more likely to develop PSBP than patients who had only a single attempt [AOR = 4.9 (95% CI: 1.63, 15.23)].

Finally, patients who had a single bone contact were 3 times more likely to develop PSBP than patients who had no bone contact [AOR = 3.1 (95% CI: 1.14, 8.45)]. Likewise, patients who had greater than a two-time number of bone contacts during the procedure were 8 times more likely to develop PSBP than patients who had no bone contact [AOR = 7.6 (95% CI; 2.60, 22.43)] (Table 3).

## 4. Discussion

One of the common complications of spinal anesthesia in clinical practice is postoperative back pain. The reduction of this pain is mandatory to increase the quality of life, expand anesthesia outcomes, and improve patient satisfaction [3, 6].

In the current study, the overall prevalence of postspinal back pain was 40.5% (95% CI: 34.0–48.4). This result was consistent with the study done in Germany (40%) [21]. However, the current finding is higher than the reports in the Republic of Korea (32%) [38]. This might be due to the fact that patients who had multiple lumbar puncture attempts (>3 times) were included in our study. During multiple lumbar puncture, there might be a cutting down of tissues and rupture of smaller blood vessels and nerves which causes back pain. This study's findings were also higher compared with the studies conducted in Europe (17%) [20] and Iran (5.8%) [39]. This discrepancy might be due to the fact that bigger sized spinal needles were used commonly in the study setup. Thus, bigger size spinal needles may damage different tissues and matters which might be subject to back pain.

In this study, the distributions of the prevalence of PSBP across postoperative days were analysed. The prevalence of PSBP was 18.1% (95% CI: 13.2, 23.1) on the first day, 11.3% (95% CI: 6.0, 12.8) on the 2^nd^ day, and 17.9% (95% CI: 8.6, 18.1) on the 3^rd^ day. This finding was in consistence with the study conducted in Iran on the 1^st^ postoperative day (16.7%) [39]. However, this finding was lower compared to the studies conducted in China (38%) [22] and Turkey (29.3%) [1] on the 1^st^ postoperative day. The reason for this difference might be due to the fact that patients who had preexisting back pain were excluded from our study.

A study conducted in Asella, Ethiopia, showed an incidence of 38, 29.9, and 31.6% of patients developed back pain on the 1^st^, 2^nd^, and 3^rd^ postoperative days, respectively [4]. This might be due to the fact that they have used big spinal needles (18–21G) and also excluded patients with preexisting back pain.

Different studies showed that PSBP mostly occurred during the use of bigger size spinal needles than smaller ones [4, 12, 17, 18]. In this study, also spinal needle size had a strong association with PSBP. Thus, patients who were given SA using a big spinal needle were 5.9 times more likely to develop PSBP than patients who received SA using small size spinal needles (AOR = 5.9 (95% CI: 1.04, 33.46). This might be due to the reason that bigger size spinal needles have a wide area penetration starting from skin to subarachnoid space including matters leading to PSBP. Another study done in Ethiopia showed that patients who had a lumbar puncture with 18 G needle were four times more likely to develop PSBP than patients who had a lumbar puncture with 21 G needle size. [17]. The possible reason for this variation might be due to the classification of spinal needle sizes. Because the study compared the 18G and 21G size spinal needles, this study classified the size of spinal needle sizes into big (18–22G) and small size (23–29G). [36].

Soft tissue damage during spinal anesthesia intervention had been studied in previous studies and found to be one of the risk factors to develop back pain following spinal anesthesia [1, 12, 18]. This might be related to the number of lumbar puncture attempts. In this study, the number of lumbar puncture attempts had a direct relationship with the development of PSBP. Thus, patients who had two attempts of a lumbar puncture during spinal anesthesia procedure were 3.6 times more vulnerable to develop PSBP than those who had one lumbar puncture attempt. On the other hand, those who had an attempt of three and more were 4.9 times more likely to develop PSBP. This might be due to repeated tissue damage and even might have nerve damage or touch which end up PSBP. Some of the studies shared that the number of lumbar puncture attempts during spinal anesthesia increases the risk of trauma and possible back pain after a surgical procedure [1, 4, 17, 40].

The likelihood to developed PSBP in patients who had single and ≥2-time bone contact during the procedure of spinal anesthesia wes 3 times (AOR = 3.1; CI: 1.14, 8.45) and 7.6 times (AOR = 7.6; 95% CI: 2.60, 22.43), respectively, more than patients who had no bone contact. This might be also due to soft tissue damage, bone scratch, and nerve injuries during bone contacts. This association is in line with the studies done in Turkey [1, 16].

Body mass index was associated with PSBP and supported by other studies [1, 41]. The present study found that patients who were overweight and obese were more likely to develop PSBP than patients with normal BMI. One of the possible reasons might be due to repeated lumbar puncture and multiple attempts during spinal anesthesia because of difficulties to identify the exact landmark in patients who had higher BMI [16, 42, 43].

Finally, as a limitation, even though this study investigates the unseen problem after spinal anesthesia at the University of Gondar Comprehensive Specialized Hospital, it includes a smaller sample size due to the small number of patient flow due to the pandemic of Covid-19. In addition, patients were followed up for only three postoperative days but they may develop postspinal back pain after they discharge from the hospital. On the other hand, the visual analogue scale score was difficult to understand for most of the study subjects to evaluate the severity of pain since most of them were illiterate.

## 5. Conclusion

This study confirms that the overall incidence of back pain is high as compared to most studies. Body mass index, the size of spinal needle, the number of attempts, and the number of bone contacts are significantly associated with the incidence of back pain following spinal anesthesia. Hence, health professionals should minimize the number of attempts and bone contacts during lumbar puncture and choose smaller spinal needles to reduce the prevalence of postspinal back pain. Finally, conducting a similar study to assess the long-term occurrence of postspinal back pain is recommended.

## Figures and Tables

**Table 1:** Sociodemographic characteristics of the study participants who underwent the surgical procedure under spinal anesthesia at the University of Gondar Comprehensive and Specialized Hospital from March to May 2020 (*n* = 215).

Variable	Frequency	Percentage (%)
Age (years)		
17–25	55	25.6
26–34	72	33.5
35–41	36	16.7
>41	52	24.2

Sex		
Female	150	69.8
Male	65	30.2

Ethnicity		
Amhara	195	90.7
Oromo	5	2.3
Other^*∗*^	15	7.0

Educational status		
Unable to read and write	63	29.3
Able to read and write	152	70.7

Occupation		
Student	18	8.4
Teacher	25	11.6
Merchant	39	18.1
Housewife	71	33.0
Farmer	43	20.0
Other^*∗∗*^	19	8.9

BMI		
18.7–25 kg/m^2^	152	70.7
25–29.9 kg/m^2^	43	20
≥30 kg/m^2^	20	9.3

ASA status		
ASA I	55	25.6
ASA II	135	62.8
ASA III	25	11.6

BMI = body mass index, ASA status = American Society of Anesthesiologist. ^*∗*^(Other ethnicity): Tigre, Wollita, Gumez, Hadire, Somali, and Agew. ^*∗∗*^(Other occupation): driver, bank accountant, secretary, unemployed, daily labor, cleaner, gardener, police, soldier, engineer, health care provider, manager, and politician.

**Table 2:** Associated factors with PSBP of the study participants at the University of Gondar Comprehensive and Specialized Hospital, Northwest Ethiopia, from March to May 2020 (*n* = 215).

Variables	Frequency	PSBP	
		Yes (%)	No (%)
BMI			
18.7–25 kg/m^2^	152	41 (27.0)	111 (73.0)
25–29.9 kg/m^2^	43	30 (69.8)	13 (30.2)
≥ 30 kg/m^2^	20	16 (80.0)	4 (20.)

Experience of anesthetist			
Student	35	16 (45.7)	19 (54.3)
1–4 years	71	28 (39.4)	43 (60.6)
>4 years	109	43 (39.4)	66 (60.6)

Previous SA			
No	151	48 (31.8)	103 (68.2)
Yes	64	39 (60.9)	25 (39.1)

Number of exposures to SA (*n* **=** 64)			
One	40	23 (57.5)	17 (42.5)
Two	20	13 (65)	7 (35)
>two	4	3 (75)	1 (25)

Type of surgery			
Obstetrics	106	47 (44.3)	59 (55.7)
Gynecology	28	5 (17.9)	23 (82.1)
Orthopedic	47	18 (38.3)	29 (61.7)
Urology	15	7 (46.7)	8 (53.3)
Other	19	10 (52.6)	9 (47.4)

Urgency of surgery			
Elective	79	30 (38)	49 (62)
Emergency	136	57 (41.9)	79 (58.1)

Duration of surgery			
<30 minutes	20	7 (35)	13 (65)
30–60 minutes	99	34 (34.3)	65 (65.7)
>60 minutes	96	46 (47.9)	50 (52.1)

Surgical position			
Supine	193	79 (40.9)	114 (59.1)
Lateral	12	4 (33.3)	8 (66.7)
Lithotomy	10	4 (40)	6 (60)

Presence of skin infiltration			
Yes	145	49 (33.8)	96 (66.2)
No	70	38 (54.3)	32 (45.7)

Number of attempts			
One	99	11 (11.1)	88 (88.9)
Two	55	31 (56.4)	24 (43.6)
>two	61	45 (73.8)	16 (26.2)

Number of bone contacts			
No contact	114	16 (14)	98 (86)
One	38	19 (50.0)	19 (50.0)
≥Two	63	52 (82.5)	11 (17.5)

Size of needles			
Big needle (18–22G)	197	85 (43.1)	112 (56.9)
Small needle (23–29G)	18	2 (11.1)	16 (88.9)

Level of interspace puncture			
L_2_-L_3_	19	15 (78.9)	4 (21.1)
L_3_-L_4_	169	55 (32.5)	114 (67.5)
L_4_-L_5_	27	17 (63)	10 (37)

Angle of the spinal needle to the skin			
Parallel	151	50 (33.1)	101 (66.9)
Perpendicular	64	37 (57.8)	27 (57.8)

Position of SA			
Sitting	206	81 (39.1)	125 (60.7)
Lateral	9	6 (66.7)	3 (33.3)

PSBP = postspinal back pain, BMI = body mass index, kg/m^2^ = kilogram per meter square. SA = spinal anesthesia, G = gauge, L = lumbar. Other procedures: foreign body removal, hernia repair, fistula repair, perianal abscess, psoas abscess, foreigners gangrene, and hemoroidectomy.

**Table 3:** Factors associated with PSBP in patients who underwent the surgical procedure under spinal anesthesia, Northwest Ethiopia, from March to May 2020 (*n* = 215).

Variables	PSBP		OR (95% CI)	
	Yes (%)	No (%)	COR	AOR
BMI				
18.7–25 kg/m^2^	41 (27)	111 (73)	1.00	1.00
25–29.9 kg/m^2^	30 (69.8)	13 (30.2)	6.2 (2.97, 13.13)^*∗*^	3.8 (1.48, 9.96)^*∗*^
≥30 kg/m^2^	16 (80)	4 (20)	10.8 (3.42, 34.29)^*∗*^	4.9 (1.19, 20.43)^*∗*^

Needle size				
Small needle (23–29G)	2 (11.1)	16 (88.9)	1.00	1.00
Big needle (18–22G)	85 (43.1)	112 (56.9)	6.1 (1.36, 27.12)^*∗*^	5.9 (1.04, 33.46)^*∗*^

Number of attempts of lumbar puncture				
Once	11 (11.1)	88 (88.9)	1.00	1.00
Twice	31 (56.4)	24 (43.6)	10.3 (4.53, 23.5)^*∗*^	3.6 (1.29, 10.42)^*∗*^
≥three times	45 (73.8)	16 (26.2)	22.5 (9.64, 52.51)^*∗*^	4.9 (1.63, 15.23)^*∗*^

Number of bone contacts during spinal anesthesia				
No contact	16 (14.0)	98 (86.0)	1.00	1.00
Once	19 (50)	19 (50)	6.1 (2.68, 14.0)^*∗*^	3.1 (1.14. 8.45)
≥Twice	52 (82.5)	11 (17.5)	28.9 (12.52, 66.94)^*∗*^	7.6 (2.60, 22.43)^*∗*^

OR = odd ratio, CI = confidence interval, COR = crude odds ratio, AOR = adjusted odds ratio, PSBP = postspinal back pain, and BMI = body mass index. ^*∗*^Significantly associated with PSBP, 1: reference.

## Data Availability

Due to ethical restrictions and privacy concerns, a dataset is available upon request from the corresponding author, Abraham Tarekegn: abrahamtm2006@gmail.com.

## Abbreviations

AOR: Adjusted odds ratio
ASA: American Society of Anesthesiologist
BMI: Body mass index
CI: Confidence interval
COR: Crude odds ratio
PACU: Postanesthesia care unit
PSBP: Postspinal back pain
SA: Spinal anesthesia
SD: Standard deviation
SPSS: Statistical package for social sciences
VAS: Visual analogue scale.
